# Learning to Care for the Mentally Handicapped
*The Burden Research Medal and Prize for 1974 have been awarded to Dr. Cowie and were presented to her at a meeting of the Burden Trustees at Stoke Park Hospital, Bristol, on 7th June, 1974, by Dr. H. J. Orr-Ewing, Chairman of the Trustees.This is Dr. Cowie's address of thanks on that occasion.


**Published:** 1974-10

**Authors:** Valerie A. Cowie

**Affiliations:** Consultant Psychiatrist, Queen Mary's Hospital, Carshalton


### Learning to care for the Mentally Handicapped*

**Valerie A. Cowie**
Consultant Psychiatrist, Queen Mary's Hospital,
Carshalton.

In his interesting account of the history of Brentry Hospital, Dr. Jancar describes the work of the Burdens who came to Bristol in 1895. This era, le fin de siècle, was a golden age for the well-endowed. The rich were very rich thanks to the bounties of the industrial revolution. The arts flourished and were well-patronised; new and interesting forms were emerging in the visual arts and in literature. But alongside this splendour, there existed in contrast much misery and squalor. To us today, the social apathy towards these ills would seem appalling. It seemed to be a generally accepted fact that the larger section of society was destined to poverty and deprivations of all kinds. It must have taken much courage to speak out against this complacency. There were, however, some brave souls who did. Among these were the Reverend Harold Nelson Burden and his wife Katherine.

When they came to Bristol in 1895, Mr. Burden was appointed chaplain to Horfield Prison. As Dr. Jancar points out in his history of their work, this marked the opening of a new chapter in the history of the care and treatment of the underprivileged, and especially of the mentally retarded. They had already worked in the East End of London and were much influenced by the work of Octavia Hill, who was a friend and disciple of Ruskin.

I will not trace the history of their achievements in Bristol; this has already been very well documented by Dr. Jancar. A signal event as regards their work for the mentally subnormal, however, was the opening of Stoke Park Colony in 1909. Much later, in 1933, this earlier work was furthered by Mr. Burden's second wife who established the Burden Mental Research Trust for the study of the causation and inheritance of normal and abnormal mentality. She also supported the foundation of the Burden Neurological Institute in 1939; its illustrious achievements have set a stamp of excellence on our national contribution to the scientific study of mental subnormality. The classical work of Professor Golla, Dr. R. M. Norman, Dr. Fraser Roberts and Dr. Grey Walter, to mention only four of the brilliant workers associated with Stoke Park and the Burden Neurological Institute, has laid solid foundations for the biological study of mental subnormality in this country. Among present workers of this group, Dr. Heaton-Ward and Dr. Jancar have added their own remarkable contributions, including their textbooks on mental subnormality which have become classics in their own right.

It is of vital importance that we nurture our scientific endeavours in mental subnormality. In recent years a schism has appeared between the scientific approach and the management approach. This gap appears to be widening. At times much acrimony is shown, and "academic" is becoming a dirty word in some quarters. But it is helpful to reflect that in reality this gap is only one of attitudes. There is no real split when it comes to the actual care of patients. For their welfare there must be close cohesion between the daily management, nursing, educational and social care on the one hand and clinical understanding and treatment on the other, which rests essentially upon the bedrock of scientific knowledge. For the sake of our patients we should close this gap which appears to exist largely in the minds of those who have been inspired to fight for territorial claims. We are all working in the same territory and although we serve in different ways, our work is complementary. If we are to continue to uphold the excellence of our tradition of work for the mentally subnormal in this country, we must work in harmony and mutual respect, paying full due to the value of each other's contributions.

Returning to the work of the early reformers, including Harold and Katherine Burden, one is struck by their overriding respect for the quality of life. This seems to have been a guiding principle in their lives and work. It leads me to a second point I would like to make. I have made this point recently elsewhere (Cowie, 1974) and I feel that all of us concerned with the welfare of the mentally handicapped and their families should pay heed to it and take positive action. My message is that we should take steps to improve and extend teaching in mental subnormality. It is a subject shamefully neglected in the undergraduate and postgraduate training of doctors, and in the teaching curriculum of general nursing. Many doctors, nurses and other workers who may be called upon at some time to treat with wisdom and discretion problems connected with the mentally handicapped and their families are virtually without training in this field. Throughout the kingdom, thousands of severely mentally handicapped patients live their lives unseen by any but those already dedicated to their care. Very many doctors have never even seen a ward for the profoundly subnormal, many of whom are multiply handicapped, and yet, at some time in their careers,

**The Burden Research Medal and Prize for 1974 have been awarded to Dr. Cowie and were presented to her at a meeting of the Burden Trustees at Stoke Park Hospital, Bristol, on 7th June, 1974, by Dr. H. J. Orr-Ewing, Chairman of the Trustees.*

*This is Dr. Cowie's address of thanks on that occasion.*

they may be called upon to take vital decisions involving misfortune of this kind. It is unreasonable to expect general improvement in the community at large for the care of mentally handicapped unless the magnitude and nature of the problem, including the quality of life of these unfortunate people are understood. The nursing and medical staff who care for a mother who has just given birth to a mongol baby, the surgeon who must exercise judgment as to the outcome of operative procedure on a child with gross central nervous system defects, the health visitor who is called upon to counsel and support the mother with a handicapped child, and those responsible for setting up hostels and other facilities for care outside hospital, all need this background of knowledge.

The negligible training in mental handicap that is given in medical, para-medical and nursing curricula is, I think, a reflection of the view that this is a second-rate subject, not without stigma even today. As long as it is regarded in this way, it will be pushed into the background, with the result that many thousands of patients and their relatives are deprived of the special consideration that they should receive. For those of us working in the field of mental subnormality, it is our urgent task to press for wider teaching of the best quality. We should make it known that mentally handicapped patients offer a rich opportunity to arrive at a full understanding of the extremes of human variance. To work with them is a privilege affording broad perspectives through which to view not only medicine but the more general problems of society and how we wish to shape the world in which we find ourselves.

### REFERENCE

COWIE, V. (1974). The role of genetics in reducing the incidence of mental disorders.
Royal Society of Health: 81st Health Congress. Papers for discussion in sessions pp. 62-64.

